# Gonorrhœal Infection in Women

**Published:** 1888-06

**Authors:** 


*Gonorrhœal Infection in Women.* By W. J. SINCLAIR, M.D. Pp. 143. London: H. K. LEWIS. 1888.

An authoritative and exhaustive treatise on this important subject was much needed in England, and Dr. Sinclair has done well in writing such a work. To deal with the subject the author brings a double fitness—firstly, in a careful and thorough personal clinical study of the whole subject; and secondly, in a minute familiarity with the writings of others. From the special point of view of literature the work is beyond all praise, being presented in clear, forcible, and graceful language.

The author does well in vigorously protesting against the erroneous and inadequate views which prevail as to the gravity of this disease. In selecting Holmes's *System of Surgery* as a mark at which to point his criticism, the author has been more kind than he need have been. The remarks in the article in Holmes's *System* are erroneous and inadequate enough to deserve sharp criticism; but we could point to articles in works scarcely less well known which are worthy only of ridicule. If Dr. Sinclair's work serves to impress on the profession the fact that gonorrhœa in women is a grave disease with a far-reaching importance, not only to the individual, but to the race, it will have fulfilled its main object. But it has didactic uses in detail as well, for it deals minutely and sensibly with all clinical and practical features. Not only is it the only monograph on the subject in the English language worthy of consideration, but it is, on its own merits, a medical work of great importance and of high merit.

